# Optimization of Ibuprofen Delivery through Rat Skin from Traditional and Novel Nanoemulsion Formulations

**Published:** 2012

**Authors:** Behzad Sharif Makhmalzadeh, Shiva Torabi, Armita Azarpanah

**Affiliations:** SchoolofPharmacy, AhvazJundishapurUniversityofMedicalSciences, Ahvaz, Iran.

**Keywords:** Ibuprofen; NSAID, Nanoemulsion, Permeability, Topical preparations.

## Abstract

The topical delivery of non-steroidal anti-inflammatory drugs (NSAIDS) such as Ibuprofen has been explored as a potential method of avoiding the first pass effects and the gastric irritation, which may occur when used orally. Ibuprofen is formulated into many topical preparations to reduce the adverse effects and simultaneously avoid the hepatic first-pass metabolism as well. However, it is difficult to obtain an effective concentration through topical delivery of Ibuprofen due to its low skin permeability. The aim of this study was to develop two types of nanoemulsions formulations and focused on the screening of Ibuprofen-loaded nanoemulsions and evaluating the influence of these types of nanoemulsions on the skin permeability of the drug. In both nanoemulsion formulations, oil was similar, but the surfactant and co-surfactant were different. The effect of independent variables on skin permeability parameters was evaluated using full factorial design. Results demonstrate that novel formulations were more effective as skin enhancer than traditional formulation. In case of the novel formulation, any increase in percentage of surfactant and co-surfactant had increasing effect on flux (Jss). On the other hand, the proportion of surfactant/co-surfactant (S/C) demonstrated reverse correlation with Jss. While, in traditional formulations, direct correlation was found between both variables, and Jss. Comparison between two types of nanoemulsion formulations revealed that, novel formulations were more effective as topical Ibuprofen carrier in contrast to traditional type due to lower amounts of surfactant and co-surfactant and less irritating effect.

## Introduction

The advantages of transdermal drug delivery rout include good patient compliance, low systemic side effect, and avoidance of the hepatic first pass effect and so better therapeutic efficacy (1). Ibuprofen, a non-steroidal anti-inflammatory drug (NSAID), can be applied in systemic treatment of various diseases such as rheumatoid arthritis, osteoarthritis and ankylosing spondylitis. The topical administration of Ibuprofen may be useful for the patients as it reduces the adverse effects; on the other hand, it avoids the hepatic first-pass metabolism and applies relatively consistent drug levels in the site of action (2). However, it is difficult to maintain effective concentrations in topical Ibuprofen delivery due to its poor skin permeability (3). In order to enhance the skin permeability of Ibuprofen, different formulations have been tested such as supersaturated solutions, mucoadhesive patches and vehicle containing non-ionic surfactants or fatty acids (4). Transdermal drug delivery of ibuprofen has been reported in various papers (5, 6). Polyoxyethylene (7) cetyl/oleyl,a non-ionic surfactant showed enhancement effect on skin permeation of ibuprofen (5). Supersaturated system has been used to enhance the penetration of ibuprofen through human skin (7). Results showed significant flux enhancement, obtained from supersaturated solution compared to the saturated solution.

Nanoemulsions are clear, thermodynamically stable, isotropic liquid mixtures of oil, water, surfactant and co-surfactant. An approximate droplet diameter of 100 nm was estimated for nanoemulsions (8). Nobody can deny the widespread application of nanoemulsions in different fields such as pharmaceutics, food, and many other industries (9). Nanoemulsion offers a promising vehicle for increasing the aqueous solubility of poorly water-soluble drugs considerably, which is usually necessary for parenteral application (10). Nanoemulsions have many advantage, for instance, enhance drug solubility, perfect thermodynamic stability, ease of manufacturing and permeation over conventional formulations that convert them to important drug delivery systems. Nanoemulsions are suitable pharmaceutical formulation for drug delivery through the skin (11). Nanoemulsion can improve transdermal delivery of lipophilic and hydrophilic compound with different mechanisms. The correlation between nanoemulsion structure or composition with its drug delivery effects have been reported and a few studies have shown that internal structure of nanoemulsion should allow free diffusion of the drug to optimized cutaneous (8). Nanoemulsion-based hydrogel formulation was applied for ibuprofen topical delivery. Nanoemulsions could increase the topical delivery of ibuprofen 5.72-30 times compare with the control (9).

This study aimed at evaluating the potential effect of traditional (made with common surfactant and co-surfactant) and novel (made with new surfactant and co-surfactant agents) nanoemulsions to effectively ibuprofen transdermal delivery through rat skin and identifying those factors that affect this transport using full-factorial experimental design and optimizing its topical delivery.

## Experimental

Ibuprofen was received from Caspian Tamin, Rashat, Iran. Isopropyl myristate was obtained from Panreac, Spain. Span 20, Tween 80, Acetonitryl, Acetic acid glacial, Chloroform, Methanol, Liquid paraffin and Ethoxylated Caster oil were all purchased from Merck, Germany. Polyethylen glycol 400 (PEG 400) was bought from Fluka, England. PEG-8 Caprylic/ Capric Glycerides (L.A.S.), Ethoxydiglycol (Transcutol CG), and Caprylocaproylmacrogolglycerides (Labrasol) were gift from GATTEFOSSE, France. All other reagents were highly commercially available.

*Animals*

Male adult Wistar’s rats (weighing 100-150 g) with the age range of 10-12 weeks were purchased from Animals Laboratory, Jundishapur University of Medical Sciences, Ahvaz, Iran. The animals were treated according to the principles of the Care and Use of Laboratory Animals and approval for the studies was given by the Ethical Committee of the Ahvaz Jundishapur University of Medical Sciences.

*Determination of solubility of Ibuprofen in different oil mixtures*

In order to find out the perfect oily phase, drug solubility was measured in isopropyl myristate, liquid paraffin and Ethoxylated Caster oil. A mixture of surfactant/co-surfactant oily phase (ratio 1 : 1 : 1) was prepared containing drug in excess, mixed for 24 h at room temperature, and then centrifuged at 3000 rpm for 10 min. Furthermore, the undissolved drug was filtered and after dilution with methanol, it was measured by HPLC (Waters, USA). Similar experiments were performed regarding the oily phase simultaneously (9). Finally, ibuprofen solubility was determined in various oils.

*Nanoemulsions preparation*

Ternary phase diagrams for determination of the components and their concentration ranges were constructed on the basis of large existence of nanoemulsion without drug or in the presence of 5% ibuprofen (12). Two main formulations including novel and traditional preparations were arranged. Major variables taking part in determination of nanoemulsion concentration range include mass ratio of surfactant (S) to co-surfactant (C), mixture weight percentage (S+C), and oily and aqueous phase weight percentage (Table 1). At the initial step, the minimum volume of S/C ratio was held in a 10 mL tube, for the purpose of investigating the surfactant weight. In the next step, the minimum volume of oily phase was added to latter mixture, and the amounts of oily phase were determined. The appearance of nanoemulsion was examined visually. Then, a turbid mixture was obtained through the application of tube shaker (Kavosh Mega, Iran), and water titration method was used as well. A volume of 20 µL water was added and the solution was cleared by repeating this procedure. Cleared solutions with low viscosities are the sign of nanoemulsions formations. The addition of water continued until observing turbidity in the sample; this is where the titration stopped, and evaluation of other formulation began. Such procedure was repeated for different oily phase at constant volume of S/C ratio (13).

**Table 1.** Different components of novel and traditional formulations.

Formulation	Surfactant	Co-surfactant	Oily phase
**Traditional**	Span20, Tween 80 (1:1 mass ratio)	PEG400	Isopropyl myristate
**Novel**	L.A.S	Labrasol	Isopropyl myristate

*Mean droplet size and distribution*

According to full-factorial design, both novel and traditional formulations were selected for mean droplet size determination through application of Particle Size Analyzer (Malvern, England). Polydispersity index was measured by Dynamic Light Scattering Spectrophotometer at 25 and 632 nm with 90 angles (14).

*Nanoemulsion characterization*

*Viscosity determination*

The viscosity of each formulation was measured by application of DV-III viscometer (Brookfield, U.S.A) at 25°C in triplicate. The measurement was performed with a number 40 spindle and the shear rate was adjusted at different rpm (14).

*Stability*

The stability of nanoemulsion formulations was evaluated through clarity and phase separation observation, in which particle size analysis was used for droplet size determination formulations at 40°C for up to 3 months. In addition, the centrifuge test was performed to determine the physical stability of nanoemulsion at 5000 rpm for 10 min (14).

*HPLC analysis*

HPLC analysis was used for ibuprofen measurement. The column was a Novapac C18 column (4 µ, 4.6 mm internal diameter × 25 cm). The mobile phase was acetate buffer (pH 4.1)-acetonitryl (pH 5.1) 50 : 50 ratio and 0.7 mL/min flow rate. Wavelength detection was considered at 264 nm. No interference of other components was detected, and all samples were filtered through an aqueous 0.45 µm pore size membrane filter before the injection.

*In-vitro permeation studies*

Full thickness of abdomen skin was separated from newly sacrificed Wistar’s rat with ether. The subcutaneous fat was completely removed and stored at freezer at -20°C. The frozen skin was kept at room temperature to carry out the permeability. The skin was mounted on the diffusion cell (MalekTeb, Iran). It must be considered that the epidermal side (skin surface area of 4.9 0.12, receiver and donor volume of 30 and 5 mL, respectively) should cover the diffusion cell completely with the face up. Both receiver and donor chambers were filled with water and remained for 16 h at room temperature, then the skins were removed, dried and their thickness was measured (13). The equilibrated diffusion cell was maintained at 37°C for 1 h. Volume of Ibuprofen nanoemulsion 5%w/v formulation (up to 5 mL) was applied as donor phase. During the experiments cells were exposed to a magnet stirring in water bath. Besides, acetate buffer 70% (pH 4.1) and acetonitryl 10% as received phase stirred permanently at 37 and 300 rpm, whereas skin temperature was 32°C. At each interval (0.5, 1, 2, 3, 4, 5, 6, 7, 8 h) a volume of 1 mL was removed from receiver phase, at the same time a new volume of 1 mL was replaced in receiver chamber. The permeability test was performed triplicate. The nanoemulsion formulations were prepared according to full-factorial design.

**Table 2:** Presentation of independent variables and levels in novel and traditional formulations

	**Variables (high level = + , low level = - )**	**Traditional formulations**	**Novel formulations**
S/C	**+**	**3:1**	**3:1**
	**-**	**1:1**	**1:1**
S + C	**+**	**40**	**50**
	**-**	**65**	**70**
W%	**+**	**18**	**20**
	**-**	**8**	**10**

*Study design for preparation of nanoemulsions and permeation*

Several parameters influence on final properties of nanoemulsions and permeation through rat skin. Full-factorial design was used concerning with 3 variables at 2 levels (Table 2). According to the table S/C ratio and percentage of sum of surfactant and co-surfactant in both novel and traditional formulations were same whereas the oily phase in novel formulation and aqueous phase in traditional formulation were different. For obtaining more documents, we evaluated ternary-phase diagrams. In the recent study, the influence of independent variables on particle size and Ibuprofen permeation parameters through rat skin considering as response was investigated (P, Tlag, Jss). The interactions’ intensity of variables on each response were estimated through simultaneous multiple regression. The optimized formulation was selected and its permeation through rat skin on the basis of surface response technique was evaluated.

*Statistical analysis*

The data was demonstrated as mean ± SD. The statistical analysis was according to paired t-test or variance analysis, followed by full-factorial design using Minitab 11 software. In order to figure out the relation between dependent and independent variables, simultaneous multi regression test was applied.

## Results

*Ibuprofen aqueous solubility*

Ibuprofen aqueous solubility was measured triplicate estimated as 0.88 0.04 mg/mL. On the other hand, in order to be confident about conformation of sink condition in Ibuprofen permeation study through rat skin, drug solubility in receiver phase containing acetate buffer 70% (pH = 4.1) and acetonitryl l10% was evaluated. The results demonstrate that Ibuprofen solubility is 325 ± 24 mg/mL (n = 3).

*Ibuprofen solubility at different oily phases*

Ibuprofen has the highest solubility in Isopropyl Myristate and secondly shows appropriate solubility in liquid paraffin and Ethoxylated Caster oil, respectively (Table 3). In addition, drug solubility in a mixture of Isopropyl Myristate accompanied by surfactant and co-surfactant was much more higher in contrast to other oily mixtures, however, addition of surfactant and co-surfactant leads to a decrease of drug solubility in the presence of Isopropyl Myristate whereas for other oils showed an increasing trend regarding drug solubility. Chen et al. obtained similar results in 2006 (17). In recent study, drug solubility was evaluated in Isopropyl Myristate, Isopropyl palmitate, Oleic acid, Ethyl oleate, and a mixture of the mentioned oils in combination with Tween 80 and propylene glycol as surfactant and co-surfactant, respectively. Results indicate that the highest drug solubility belongs to oleic acid primarily then Isopropyl Myristate, while addition of surfactant and co-surfactant to isopropyl myristate results in decrease of drug solubility. Ibuprofen solubility in Isopropyl Myristate equals 0.16 ± 0.015 g/mL

**Table 3:** Demonstration of Ibuprofen solubility at different oil: surfactant: co-surfactant mixtures (ratio 1 : 1 : 1).

Oil	Solubility (g/mL)
**Isopropyl Myristate**	0.196 ± 0.029
**Liquid paraffin**	0.138 ± 0.019
**Ethoxylated Caster Oil**	0.112 ± 0.014
**Isopropyl myristate:Tween80:Peg400**	0.176 ± 0.025
**Liquid paraffin:Tween80:PEG400**	0.155 ± 0.017
**Ethoxylated Caster Oil:Tween80:PEG400**	0.124 ± 0.010

*Phase studies*

**Figure 1:**
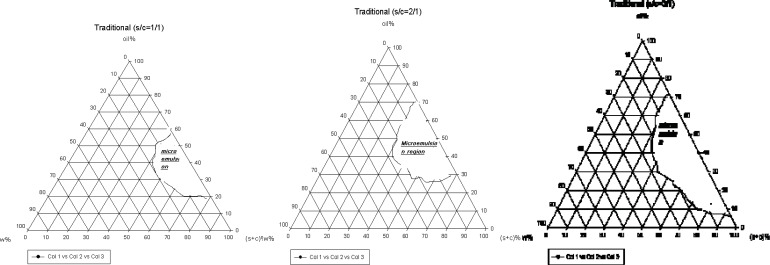
The pseudo-ternary phase diagrams of S/C system at the 1 : 1, 1 : 2 and 1 : 3 weight ratios for traditional formulations at 25°C.

The systems were composed of Tween 80: Span 20 as surfactant and PEG 400 as co-surfactant. The phase diagrams were used to investigate the nanoemulsion regions. The pseudo-ternary phase diagrams were constructed in various weight ratios of S/C (Figure 1).

The translucent nanoemulsion region can be observed in phase diagrams. The diagrams indicate that a rise in S/C ratio leads to a more extensive nanoemulsion regions and presence of much more water in the structures. All the nanoemulsions contain 4-20% water, about 30-70% mixture of surfactant and co-surfactant and 25-64% oil. However, traditional formulations contain low amounts of water and are w/o type. Two types of non-ionic surfactant were used for preparation of traditional formulations; a combination of non-ionic and ionic surfactant is essential for developing the nanoemulsion region (16).

**Figure 2:**
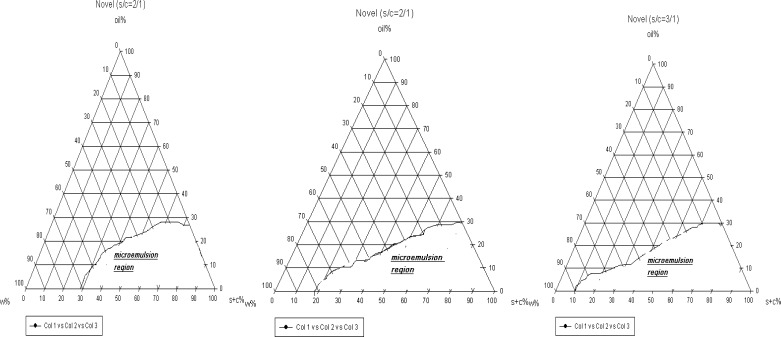
The pseudo-ternary phase diagrams of S/C system at the 1 : 1, 1 : 2 and 1 : 3 weight ratios for novel formulations at 25°C.

For preparation of novel formulations LAS and Labrasol were used as surfactant and co-surfactant, respectively. The nanoemulsion regions are wider in novel preparation in contrast to traditional (Figure 2). In addition, in both novel and traditional formulations the ranges of nanoemulsion concentrations developed, while we prepare 1 : 3 ratio of S/C in comparison to 1 : 1. In novel formulations 5-88% water, 1-30% oil and 10-85% combination of surfactant and co-surfactant exist consequently as high amounts of water can be held in nanoemulsion construction. From this aspect novel preparations are more economic and lower irritation effect observes due to the less content of surfactant. In such formulations nanoemulsions are of o/w type, since they contain great amounts of water and observation of micellar constructions is predictable (16).

According to the full-factorial design, 8 novel and traditional formulations were selected and the characterization of each formulation was examined separately before the permeation study through the rat skin.

*Characterization studies*

*Viscosity*

Among traditional formulations 1, 2, 5, 7 have the highest viscosity, but 3, 4, 6, 8 formulations showed low degree of viscosity.

The same experiments were performed concerning novel preparations. Formulations 1, 2, 5 containing a great amount of surfactant and co-surfactant, showed a high value of viscosity, although formulation 7 did not follow the similar trend. Comparison between novel and traditional preparations indicates that traditional formulations have much more attitude to be dispersed rather than the novels. Apart from that the percentage of surfactant and co-surfactant in traditional formulations has more effect on viscosity in contrast to novel ones. We expect that except for the mentioned parameters, other factors such as oil percentage and S/C ratio affect viscosity changes in novel formulations. However, the viscosity was not significantly higher in traditional formulations than novel ones (p = 0.085), (Table 4).

*Mean particle size*

All formulations were concerned in two categories for traditional preparations. The first group includes the ones with mean particle size below 25 nm that prepared by a ratio of 1 : 1 S/C including formulations number 5, 6, 7, 8 and the second has the mean particle size above 30 nm consist of a great ratio of S/C, including formulations number 1, 2, 3, 4. In addition, the difference between mean particle size in two categories is significant (p = 0.005). It seems that the amounts of co-surfactant plays an important role in mean particle size variations; on the other hand, PEG 400 in traditional preparations as a co-surfactant forms a film around dispersed and leads in a decrease of particle size. As observed, the polydispersity index demonstrates uniformity of particle size in all formulations.

Similar tests were carried out regarding novel preparations. All the novel formulations showed mean particle size below 15 nm except formulations 2, 5, 7, 8, thus great consumption of co-surfactant in S/C ratio and high percentage of surfactant and co-surfactant leads to decrease in particle size. In conclusion, the particle size in novel formulations is significantly less than traditional (p = 0.0016); thus, LAS and Labrasol components form a film round the particles which cause decrease in interface forces and more small particle size. Such investigations are consistent with phase studies in which a greater nanoemulsion region belongs to novel formulations. It should be considered that both traditional and novel formulations showed appropriate uniformity of particles (Table 4).

**Table 4:** Characterization of traditional and novel formulations (mean ± SD, n = 3). Similar experiments were performed for novel formulations. The results illustrate that in novel preparations, both S/C and percentage of surfactant and co-surfactant parameters affect mean particle size, although this relation is not completely significant (p = 0.135 and p = 0.085, respectively).

Polydispersity index		Mean particle size(nm)		Viscosity (cps)		Formulation No.
Novel	Traditional	Novel	Traditional	Novel	Traditional	
**0.24 ± 0.011**	0.3 ± 0.01	19 ± 0.5	30 ± 1.4	50 ± 1.7	60 ± 3.1	1
**0.19 ± 0.008**	0.25 ± 0.009	13 ± 0.7	30 ± 1.3	48 ± 2.3	56 ± 2.3	2
**0.15 ± 0.005**	0.31 ± 0.013	25 ± 0.9	37 ± 1.0	42 ± 1.5	45 ± 1.6	3
**0.21 ± 0.007**	0.28 ± 0.012	20 ± 0.8	35 ± 1.4	43 ± 1.2	41 ± 1.1	4
**0.28 ± 0.009**	0.19 ± 0.014	10 ± 0.4	22 ± 0.7	47 ± 2.0	57 ± 2.2	5
**0.25 ± 0.006**	0.2 ± 0.008	18 ± 0.6	23 ± 1.1	40 ± 1.8	40 ± 2.3	6
**0.12 ± 0.004**	0.22 ± 0.007	15 ± 0.3	19 ± 0.4	41 ± 1.6	53 ± 2.8	7
**0.15 ± 0.006**	0.22 ± 0.011	16 ± 0.2	25 ± 0.9	38 ± 1.4	44 ± 1.9	8

Different regression analyses were used for evaluation of independent variables on nanoemulsion particle size. Regarding traditional formulation results indicate that S/C (p = 0.001) and S+C % significantly affect mean particle size, while S/C ratio has the highest effect. However, surfactant and co-surfactant percentage have diverse relationship with particle size changes.

It should be considered that for novel preparations the percentage of surfactant and co-surfactant critically influence mean particle size, whereas for traditional formulations the most important factor is S/C ratio. The results show that in case of novel preparations, the interaction of three variables magnificently affects mean particle size variations.

*Stability*

Both novel and traditional formulations did not show any change of phase separation during 6 months. Amounts of Ibuprofen in different formulations at 40°C for 3 months were 94 ± 3% (n = 8) and 97 ± 2% (n = 8) for traditional and novel formulations, respectively. However, traditional formulations showed a little instability at 40°C which is due to thermal sensitivity.

*Ibuprofen effect on nanoemulsion structure*

In order to evaluate the effect on nanoemulsion formulation Ibuprofen was added to the formulation in two steps: first, the addition of drug was carried out to oily phase containing surfactant and co-surfactant after the nanoemulsion was formed by water titration. In the second procedure, Ibuprofen was added after formation of nanoemulsion. The results illustrate that traditional formulations were transparent in first procedure although they became initially turbid in the second procedure; nevertheless, novel preparations were transparent in both procedures.

Traditional formulations are mostly w/o nanoemulsions since after the addition of Ibuprofen, turbidity of formulation is observed as a result of particle size growth; however, the drug causes reformation of nanoemulsion as it establishes a surfactant film (17). Since novel formulation contains bicontineous structure, surfactant film has the capacity to avoid particle size growth and maintains the formulation in nanoemulsion region.

*Ibuprofen nanoemulsion permeation studies from rat skin*

Different parameters were investigated through permeation studies, including flux (Jss), permeability coefficient (p), lag time (Tlag) and diffusion coefficient (D). The linear slope of accumulative drug amount against time curve is considered as Jss. P was obtained through Jss = P C, in which C represents the drug concentration in donor phase and has values of 0.88 mg/mL and 50 mg/mL for control and nanoemulsions groups, respectively. On the other hand, by crossing the steady state section of permeation profile on the horizontal axis, D parameter can be easily found.

Tlag = h^2^ / 6d (Equation 1)

Since h demonstrates skin thickness and practically does not show the real pathway for drug permeability, the diffusion coefficient is defined as appearance D. However, confirmation of sink condition was necessary for calculation of Jss and p parameters; therefore the maximum concentration at receiver phase was less than 3% of drug solubility. Laplace transformation technique was used according to finite and infinite dose to obtain less error in calculations. In this technique because of estimation of momentary velocity the error comes to its lowest level (16). For simulation of skin into normal condition, skin samples were hydrated from approximately 10 to 20%. The hydration level was checked by gravimetric method. To that end, the weights of sample before and after hydration period (over night) were measured and hydration level estimated by equation no 2 (19). Samples thickness were 340 ± 45 µ (n = 35).

Hydration level = (weigh after hydration- weight before hydration) / weight before hydration (Equation 2)

*Traditional formulation*

In this experiment, the permeability parameters were calculated according to cumulative amounts of drug by application of full-factorial design (Table 5).

The relation between independent variables and P demonstrated that S/C ratio and (S+C) % significantly affect P, as an increase in two mentioned factor leads to elevation of P (p = 0.005 and p = 0.009).

The percentage of aqueous phase did not significantly affect Jss (p = 0.085). Surfactant deforms the skin structure, and simultaneously causes an increase in P; on the other hand, through an increase in surfactant and co-surfactant a decreasing trend in solubility of ibuprofen occurs and promotion of drug thermodynamic activity leads to P elevation. Comparison between P parameter in control group and nanoemulsions indicate that formulation 1 and 2 are significantly different (p < 0.05). The same regression analysis was performed concerning with Tlag parameter.

The relation between S/C and (S+C) % with Tlag was significant (p = 0.05); an increase in factors of interest leads to decrease in Tlag, which represents the effect of surfactant on skin structure. Comparison between control group and nanoemulsions illustrates that formulation 1, 2, 4 could result in a significant decrease of Tlag.

Formulations number 1, 2, 4, 6, 7, on the other hand show higher value of diffusion coefficient in comparison to control group (p < 0.05).

The relation between independent variables and D parameter demonstrates that any of the independent variables significantly influence D parameter. Meanwhile, we compared the results obtained from D studies and simultaneously P parameter that showed the major effect of variables on P parameter, which is due to the influence on distribution coefficient between skin and formulation. Eventually, surfactant and co-surfactant facilitate the distribution of drug through skin. Considering the fact that the relation between the effect of surfactant and co-surfactant was insignificant with D parameter, the influence of surfactant mixture is mainly focused on skin ability for solubility of Ibuprofen.

*Novel formulation*

Similar experiments were carried out for novel preparations (Table 6). The results illustrate that three variables significantly affect P (p < 0.05). It should be noted that the relation between S/C ratio and oil phase percentage with P is diverse although (S+C) %, which directly affects the P, and subsequently, higher consumption of surfactant in the formulation results in the promotion of P and this is diversely true for traditional formulations.

**Table 5:** Presentation of different parameters interfering with Ibuprofen permeation through rat skin for traditional formulations (mean ± SD, n = 3).

**Formulation**	**Jss (μg/cm**2**.h)**	**P (cm/h)**	**D (appearance) (cm**2**/h)**	**Tlag (h)**	**Full-factorial state**
**Control**	83.5 **± **6.3	128	0.0069 **± **0.0008	2.6 **± **0.17	-
**1**	137 **± **7.9	2.75 **± **0.29	0.012 **± **0.005	1.86 **± **0.12	+++
**2**	133 **± **9.4	2.66 **± **0.31	0.01 **± **0.007	1.97 **± **0.14	++-
**3**	120 **± **4.5	2.4 **± **0.18	0.0066 **± **0.0005	2.39 **± **0.25	+--
**4**	123 **± **6.2	2.46 **± **0.21	0.0097 **± **0.0008	2.27 **± **0.18	+-+
**5**	118 **± **7.7	2.36 **± **0.17	0.0076 **± **0.0004	2.32 **± **0.15	-++
**6**	112 **± **5.1	2.24 **± **0.12	0.0097 **± **0.0006	2.44 **± **0.1	--+
**7**	115 **± **3.9	2.3 **± **0.21	0.011 **± **0.0003	2.35 **± **0.19	-+-
**8**	107 **± **4.3	2.14 **± **0.16	0.0064 **± **0.0006	2.4 **± **0.19	---

It could be pointed that the above results are closely connected to phase behaviors, thus the higher amounts of Labrasol in novel formulations may form bicontineous structures and the actual development of skin permeability (19). In traditional formulations, PEG 400 plays a key role as co-surfactant. On the other hand, PEG 400 demonstrated retardant effect on drug release and loading in the skin. The retardation of percutaneous absorption by PEG was reported for diethyl-mtoluamide (DEET) (20). This study indicated that laurocapram as percutaneous penetration modifiers enhanced DEET permeation in propylene glycol, but retarded in PEG400. Retardation effect of PEG attributed to strengthening of lipid-protein complex and organization of stratum corneum lipids by increased H-bonding. These finding can be considered for present study. The amounts of oily phase in the formulation make the P parameter decrease; in other words, Jss has a higher value in nanoemulsions in comparison with control group, which may lead to a conclusion that it is an indicator of absorption enhancer in novel nanoemulsions (p < 0.05).

Several investigations were carried out to figure out the influences of independent variables on Tlag and D parameters for novel formulations.

According to the regression analysis, it was found out that S/C ratio was direct and could significantly affect Tlag (p = 0.018). An increase in surfactant amount leads to the decrease of Tlag by forming bicontineous structures. Studies demonstrate that this fact accelerates the permeation of drug in nanoemulsion, which mainly affects Tlag parameter (20). As a matter of fact, all novel formulations significantly decrease Tlag and are capable of accelerating drug onset of action.

A similar pattern was observed in D parameter studies. S/C ratio is the only factor that is in diverse and significant relation to D parameter (p = 0.017); from another aspect the higher amounts of co-surfactant in formulation results in D parameter elevation. The co-surfactant in novel formulation can promote drug diffusion as well as changing diffusion coefficient. On the basis of equation, P parameter will be changed by K and D parameters, showing the fact that S/C ratio diversely affects P and D. It seems that the enhancing effect of novel formulation on P was due to D. The incredible role of Labrasol in novel formulations results in promotion of D parameter in contrast to control group (p < 0.05).

Comparison between the effects of two formulations on permeability parameters indicated that maximum enhancement of Jss for novel and traditional formulations were 2.5 and 1.65, respectively. It seems that the types of oil phase and co-surfactant and s/c ratio and (s + c) % are very important variables that influence ibuprofen permeation through rat skin. The effect of nanoemulsion on ibuprofen permeation through porcine skin was also reported (8). In this study various formulations were made by ethyl oleate as oil phase, Tween 80 as surfactant and propylene glycol as co-surfactant. Results showed nanoemulsions increased ibuprofen permeation rate 5.72-30 times. The internal structure of nanoemulsion plays a critical role on cutaneous delivery, showing that ibuprofen release from nanoemulsion in present study is lower than nanoemulsion made by ethyl oleate, Tween 80 and propylene glycol.

**Table 6:** Presentation of different parameters interfering with Ibuprofen permeation through rat skin for novel formulations (mean ± SD, n = 3)

**Full-factorial state**	**Tlag (h)**	**D (appearance) (cm** ^2^ **/h)**	**P (cm** ^2^ **/h)**	**Jss (μg/cm** ^2^ **.h)**	**Formulation**
**-**	2.76 ± 0.185	0.0075 ± 0.0007	135 ± 6 7.5	78.3 ± 6.54	Control
**+++**	1.35 ± 0.14	0.0123 ± 0.001	3.02 ± 0.27	151 ± 12.8	1
**++-**	1.27 ± 0.09	0.015 ± 0.0009	3.38 ± 0.2	169 ± 10.3	2
**+--**	1.33 ± 0.12	0.012 ± 0.0013	2.95 ± 0.28	147 ± 14.5	3
**+-+**	1.66 ± 0.11	0.0101 ± 0.0008	2.85 ± 0.17	144 ± 7.6	4
**-++**	0.97 ± 0.066	0.025 ± 0.0017	3.5 ± 0.33	175 ± 15.8	5
**-++**	1.18 ± 0.006	0.017 ± 0.001	3.4 ± 0.36	170 ± 18.11	6
**-+-**	1.05 ± 0.008	0.0186 ± 0.0015	3.9 ± 0.33	195 ± 16.6	7
**---**	1.2 ± 0.075	0.02 ± 0.0016	3.65 ± 0.35	180 ± 16.1	8

*Skin permeation optimization*

In order to achieve a formulation with optimized permeation through rat skin, active variables which significantly influence the response were used and skin permeation were performed with these active variables using a central composite design (CCD). For this purpose, additional formulations were provided with 23 factorial design and central points (four formulations) and the effect of active variables on skin permeation parameters were evaluated.

In traditional preparations, D and P parameters were eliminated from the field of study since they were not significantly related to the independent variables. Thus both Jss and Tlag parameters were considered appropriate for the purpose of formulation optimization. Results indicate no effect of water percentage on both Tlag and Jss factors, which makes the role of S/C ratio and (S + C) % more apparent in nanoemulsion optimization. Since aqueous phase content and Jss are not significantly related to the equation 3, (S + C) % and S/C ratio demonstrate direct and significant correlation with Jss.

Jss = 2.1 + 0.29 (S+C)% + 5.28 (S/C) (Equation 3)

The modified equation for Tlag was as follow:

Tlag = 3.14-0.01 (S+C)% - 0.123 S/C (Equation 4)

In contrast to Jss, any increasing in the amount of (S + C) and (S/C) will decrease the Tlag.

**Table 7:** Properties of central composite formulations accompanied by Jss and Tlag in traditional preparation.

**Formulation No.**	**Independent variables**		**Experimental parameters**		**Calculative parameters**		
	**S/C**	**(S+C)%**	**J**_ss_	**T**_lag_	**J**_ss_	**T**_lag_	
**1**	2	55	138.8	2.4	117		2.34
**2**	2	65	122	2.3	120.9		2.24

**Table 8:** Central composite characterizations for novel formulations.

**Formulation No.**	**Independent variables**			**Experimental parameters**		**Calculative parameters**	
	**S/C**	**(S + C)%**	**Oil%**	**J**_ss_	**T**_lag_	**J**_ss_	**T**_lag_
**1**	2	60	15	119	1.22	116	1.14
**2**	2	70	15	190	1.12	193	1.05

In order to verify the above equation, two different formulations were prepared, thus Tlag and Jss were estimated and compared with each parameter calculated with equation. The optimized formulations concerning the traditional preparation contained 65% of (S+C) with S/C 1:3 ratio at minimum and maximum values of Jss and Tlag, respectively (Table 7).

In the recent study, the above investigations were analyzed regarding novel preparations as well. The results indicate that both Jss and P parameters were significantly related to independent variables, whereas Tlag with S/C and (S+C) % as D parameter with S/C followed such a relationship.

According to the acquired results, the optimized novel formulation contains minimum S/C ratio apart from maximum of (S+C) % with 1 and 70% values, respectively. In order to verify the above equations, two different formulations were prepared, thus Tlag and Jss were estimated and compared with each parameter calculated with equation. (Table 8). It should be pointed that such a formulation possesses minimum Tlag in contrast to a maximum value of Jss with optimized permeability through rat skin.

## Conclusions

Phase diagrams indicated more extensive nanoemulsion region with a rise in S/C. Consequently, wider nanoemulsion region was provided using novel formulation. Particle size in novel formulations was significantly less than traditional ones. It seems that LAS and Labrasol produced elastic film round the particles. In permeation experiments, the correlation with Jss and S/C ratio was directly and diversely for traditional and novel formulations respectively. It suggests that the effect of co-surfactant on Jss was more effective than the traditional one. It seems that PEG as cosurfactant in traditional formulation may plays as retardant. However, novel formulation demonstrates better formulation for ibuprofen transdermal delivery. Novel formulations produced smaller particle sizes and higher Jss. Jss in novel formulations controlled S/C ratio, oil% and (S+C) % in a way that any increase in co surfactant increased Jss. In conclusion, the kind of ingredient plays a crucial role in the properties of nanoemulsions and ibuprofen permeability through skin.
